# The Effect of Different System Parameters on the Movement of Microbial Cells Using Light-Induced Dielectrophoresis

**DOI:** 10.3390/mi15030342

**Published:** 2024-02-29

**Authors:** Devin Keck, Suma Ravi, Shivam Yadav, Rodrigo Martinez-Duarte

**Affiliations:** Multiscale Manufacturing Laboratory, Department of Mechanical Engineering, Clemson University, Clemson, SC 29634, USA

**Keywords:** microbial manipulation, motion, light, electric, particle, dielectrophoresis

## Abstract

The manipulation of single particles remains a topic of interest with many applications. Here we characterize the impact of selected parameters on the motion of single particles thanks to dielectrophoresis (DEP) induced by visible light, in a technique called Light-induced Dielectrophoresis, or LiDEP, also known as optoelectronic tweezers, optically induced DEP, and image-based DEP. Baker’s yeast and Candida cells are exposed to an electric field gradient enabled by shining a photoconductive material with a specific pattern of visible light, and their response is measured in terms of the average cell velocity towards the gradient. The impact on cell velocity when varying the shape and color of the light pattern, as well as the distance from the cell to the pattern, is presented. The experimental setup featured a commercial light projector featuring digital light processing (DLP) technology but mechanically modified to accommodate a 40× microscope objective lens. The minimal resolution achieved on the light pattern was 8 µm. Experimental results show the capability for single cell manipulation and the possibility of using different shapes, colors, and distances to determine the average cell velocity.

## 1. Introduction

The physical manipulation of micrometer-sized particulates finds application in multiple technologies, from microrobots to cell analysis [1,2,3,4]. Different ways to gain control of the targeted particle in time and space exist and can be classified as direct contact, as in physical tweezers, or contactless, thanks to the induction of a force through the application of a field, such as magnetic or electric fields [5]. Although the use of magnetic fields is widely reported for non-contact manipulation [6], it is limited by the need for magnetic particles. On the other hand, electric fields can enable manipulation of a larger variety of particles. Of particular interest when using electric fields is the dielectrophoresis phenomena, or DEP, which results in particle movement due to the interaction between an electric field gradient and the electric dipole that is induced on a particle. In contrast to electrophoresis, DEP does not require the particle to be electrically charged, only to be electrically polarizable, and it requires a non-uniform electric field [7].

Light-induced DEP (LiDEP) is an alternative to the conventional approach of using an array of microelectrodes fabricated with a fixed geometry and specific gaps between electrodes to establish the required field gradient for DEP. Also known as optically induced dielectrophoresis (ODEP) [8,9,10,11,12], image-based dielectrophoresis (iDEP) [13], and optoelectronic tweezers (OET) [14,15,16], LiDEP devices feature a layer of photoconductive material instead of electrodes patterned from a thin film. A pattern of light is then used to excite the photoconductive material and thus create the desired field gradient. Since the pattern of light can be easily changed during an experiment, LiDEP allows for the dynamic control of the electric field gradient during an experiment, making this technology a preferred way to manipulate particles in arbitrary trajectories at will. LiDEP was first described by Chiou in 2003 with the successful manipulation of 25 µm polystyrene beads and *E. coli* bacteria [17]. Shortly after, their work continued by demonstrating the manipulation of many particles and cells [18]. Since 2005, many groups have continued this work. For example, LiDEP has been used for the high-purity manipulation and isolation of circulating tumor cells (CTCs), the manipulation of HeLa and Jurkat cells in high-conductivity media, and the manipulation of white blood cells, red blood cells, and yeast [8,9,10,19,20,21,22]. Indeed, LiDEP has become an attractive manipulation tool since it is a noncontact, noninvasive technique that offers the flexibility of trapping, transporting or isolating cells through static or mobile light patterns that facilitate the control of a nonuniform electric field. The ability to continually change the geometry and location of these light patterns provides a significant advantage over traditional DEP manipulation techniques by allowing continuous control of the electric field gradient in both space and time. LiDEP is also often compared to the use of optical tweezers due to their similar ability of manipulating cells using a light pattern, however LiDEP has the added advantage of producing motion forces equivalent to or greater than optical tweezers with a much lower power. This is due to the fact that LiDEP utilizes the light only as the stimuli for the photoconductive layer, which is interfaced to an external power supply, whereas optical tweezers utilizes light directly as the mode of manipulation. Manipulation of cells with LiDEP has been reported at optical intensities 100,000 times lower than the intensity required for optical tweezers, allowing the light patterns to be projected over an area 500 times greater than optical tweezers [23]. Hence, the required light patterns can be enabled by common image projectors as opposed to a high intensity laser [13,18].

In this work, we characterize the effect of different LiDEP variables on the movement of selected microbial cells. Parameters studied include the shape of the light pattern, the color of the pattern, and the space between the cell of interest and the light pattern intended to use for cell manipulation. We aim to characterize these relationships in order to enable the precise manipulation of targeted particles. 

## 2. Theoretical Background

Dielectrophoresis (DEP) refers to the movement of electrically neutral but polarizable particles, because of dipolar polarization when exposed to a non-uniform gradient of an electric field. Two particular behaviors can be displayed by the particle when exposed to the required field gradient: positive DEP when it moves towards high-gradient intensity, and negative DEP when it moves away. Briefly, such behavior can be deducted from Equation (1), which is a widely used expression for the DEP force exerted on a spherical particle when exposed to a gradient ∇ of an electric field *E*:

*F_DEP_* = 2π*r*^3^*ε_m_*[*K*(*ω*)]∇(E^2^) (1)

where *ε_m_* is the permittivity of the suspension media, *r* is the radius of the particle, and *K*(*ω*) is the real part of the Clausius–Mossotti factor given by Equation (2),

(2) $$K(\omega )=\frac{\left(\varepsilon_{p}-j\frac{\sigma_{p}}{\omega })-(\varepsilon_{m}-j\frac{\sigma_{m}}{\omega }\right)}{\left(\varepsilon_{p}-j\frac{\sigma_{p}}{\omega })+2(\varepsilon_{m}-j\frac{\sigma_{m}}{\omega }\right)}$$

where *σ_p_* and *σ_m_* are the conductivities of the particle and the medium, respectively, and *ω* is the angular frequency of the polarizing electric field. Hence, negative values of *K*(*ω*), and negative DEP, result when the particle is less electrically polarizable than the suspending media; while attraction to the field gradient, or positive DEP, results otherwise. In this present work, we use positive DEP to attract a targeted particle to a field gradient enabled by light and we use particle velocity to characterize the strength of the DEP force. The velocity of a particle that is approximated as spherical and is suspended in a fluid with viscosity η under the influence of a DEP force u_DEP_ can be estimated by equating the DEP force with the Stokes’ drag force, Equation (3), and assuming all other forces in the system of interest are negligible as previously reported by many authors,

(3) $$u_{\mathrm{DEP}}=\frac{F_{DEP}}{6\pi \eta r}$$

Thus, such a direct relationship between the particle velocity and the DEP force exerted on a given particle dictates that observation of a higher velocity corresponds to a stronger DEP force. 

## 3. Materials and Methods

### 3.1. Experimental Setup

**Figure 1:**
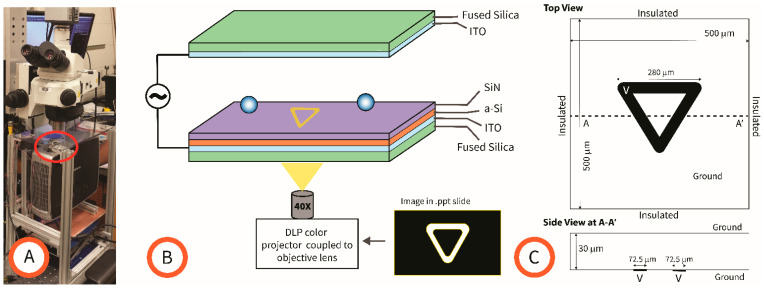
(**A**) A picture of the LiDEP setup showing the projector used to generate the pattern and the upright microscope used for visualization of the experiment. The red ellipse indicates the positioning of the experimental device in the system. (**B**) Schematic of the experimental device. The bottom electrode featured a stacking of electrically conductive indium tin oxide ITO; photoconductive amorphous silicon a-Si; and a passivating layer of silicon nitride (SiN) on top of a fused silica substrate. The top electrode featured a single layer of ITO on top of fused silica. Stretched parafilm was used to create the experimental chamber. (**C**) Top and side views of the computational model showing dimensions, values assigned to boundaries, and the modeled pattern (in black). Voltage is applied to the pattern, a triangular one in this case.

While there are multiple ways of inducing the required field gradient for DEP [24], in this work we emphasize the use of photoconductive materials and light patterns to enable LiDEP. The experimental setup is shown in Figure 1. The important blocks in the system are the generation of a light pattern, the photoconductive device, and the experimental chamber. A crucial element in LiDEP is a photoconductive layer, or a material that becomes electrically conductive when exposed to light of a specific wavelength, such as the amorphous silicon (a-Si) used here. A typical LiDEP platform features two parallel electrodes separated by a given distance, and at least one of them coated with a-Si. Since Indium Tin Oxide (ITO) is transparent to light wavelengths adequate to excite a-Si, such electrodes are usually made out of ITO. In such a setup, the non-uniform electric field gradient can easily be generated by electrically polarizing the ITO electrodes and shining a pattern of light on the a-Si. In comparison to more conventional DEP methods, where electrode patterns and arrays are fabricated out of conductive materials, LiDEP allows for dynamic control of the field gradient configuration since the photoconductive layer can be seen as a blank canvas where one can continuously “draw” electrodes using light.

Light patterns used in this work included circles and selected geometric shapes, and all were drawn in Power Point (.ppt, Office 365) slides in front of a black background. The color of the pattern was varied according to the experiment conducted. Colors used were codified in RGB and included white (255, 255, 255), red (255, 0, 0), green (0, 255, 0), blue (0, 0, 255) and yellow (255, 255, 0). The movement of the light pattern was controlled by flipping through a deck of slides in presentation mode at an arbitrary speed. A modified DLP (Digital Light Processing) color projector was used to generate and shine the light pattern on the a-Si (Figure 1). Specifically, the magnification lens in an InFocus (Tigard, OR, USA) IN24 DLP projector was replaced with a Nikon (Tokyo, Japan) 40× microscope objective lens (LWD Plan Infinity Objective lens, Working Distance = 3.71 mm, Numerical Aperture = 0.60) after mechanically modifying the plastic frame of the projector and installing a threaded ring (Thorlabs, Newton, NJ, USA). The wavelength and relative intensities of the light projected by the different colors used in this work were measured with a spectrometer (USB400, Ocean Optics, Ostfildern, Germany) by shining an entire field of view, i.e., a full Power Point slide, of a given color directly onto the detector from a set distance. The intensities were calculated by integrating the area under the plots of wavelength versus counts using the trapezoidal method of integration implemented in a MATLAB code (version R2023b). To report the intensity of each projection in absolute terms, a power meter (PM120VA/PM400k1, Thor Labs, Newton, NJ, USA) was utilized to measure the absolute intensity of the red projection and used to normalize the measurements of all other colors. 

The electrical setup, including the photoconductive electrode, is illustrated in Figure 1B. The top ITO electrode was a 100 nm-thick layer fabricated on a fused silica substrate using a PVD75 RF sputterer (Kurt J Lesker, Jefferson Hills, PA, USA). ITO was selected as the top electrode due to its transparency to the wavelength of light used in these experiments and to enable visualization of the experiment with an upright microscope (Nikon Eclipse LV100). The bottom electrode featured a photoconductive layer of a-Si on top of an ITO layer. Similar to the top electrode, the fabrication process for the bottom electrode began with a PVD75 RF Sputterer deposition of a 100 nm layer of ITO onto a fused silica wafer. Next, a Unaxis (St. Petersburg, FL, USA) PECVD machine was used to deposit 1 µm of a-Si. Lastly, an Oxford Instruments (Bristol, UK) PECVD PlasmaLab 80 plus machine was utilized to deposit a 10 nm-thick passivating layer of Silicon Nitride (SiN) on top of the a-Si. Indium solder was used to wire the top and bottom electrodes to electrical leads that facilitated connection to a function generator (BK Precision 4040B, Yorba Linda, CA, USA).

The experimental chamber was fabricated out of paraffin wax film (parafilm^®^). To this end, parafilm was manually stretched to a thickness within a range of 10−30 µm (as measured with a caliper) and then manually cut with a blade into a ring that was used as a spacer between the top and bottom electrodes. No clamping was necessary since flow was not required during experiments. This simple approach enabled quick assembly of the experimental setup and easy cleanup after experiments. 

### 3.2. Computational Model

The parameter ∇(*E*^2^) for shapes implemented during experiments, including the triangular pattern shown in Figure 1, was modeled in COMSOL 6.0.2 running in a PC featuring an Intel 13th generation i9 processor with an NVIDIA GeForce RTX 4060 graphics card and 64 Gb of RAM. The polarization voltage V was swept from 5 to 10 V in increments of 2.5 V. The media was modeled as water with conductivity of 0.002 S/m. 

### 3.3. Cell Culture and Sample Preparation

*Saccharomyces cerevisiae* (Sigma-Aldrich 51475, St. Louis, MO, USA), *Candida albicans* (ATCC 18804), and *Candida glabrata* (ATCC2001) were cultured in dynamic conditions at 37 °C and 215 rpm in yeast malt broth (YMB) and passed twice a week to maintain a healthy culture. To prepare the experimental sample, 50 µL of 3-day old cell culture were mixed with 2.5 mL of an optimized DEP buffer solution composed of 8.6 wt% sucrose, 0.3 wt% dextrose and 0.1 wt% bovine serum albumin to achieve a concentration of around 10^5^ cells/mL. The electrical conductivity of this DEP buffer solution was 0.002 S/m (Oakton PC700 Benchtop Meter, Charleston, SC, USA). Cells were then pelleted through centrifugation at 5000 rpm (Labnet Hermle Z200A, Edison, NJ, USA) for 5 minutes and then resuspended into fresh DEP buffer solution. This centrifugation and resuspension protocol was repeated three times to ensure the complete removal of any remaining YMB culture media. Although Candida cells have significant clinical relevance [25,26], their use here was limited to dielectric particles of a biological nature to facilitate the study of the performance of the LiDEP platform on bioparticles. The reader is directed to other works regarding DEP of Candida as a potential tool for healthcare diagnostics [27,28]. 

### 3.4. Experimental Protocol and Data Analysis

Once assembled, ~10 µL of experimental sample was manually pipetted in and the chamber was topped with the top ITO electrode. All experiments were performed under an electrical stimuli with an applied voltage of 10 V_pp_ and a frequency of 250 kHz. These values were previously chosen based on the observation of a strong attractive response from the cells of interest [27] and the lack of electrothermal effects previously observed by other groups [29]. As detailed in Section 2, the DEP response for all characterizations presented in this section were measured based on cell velocity. A higher cell velocity is indicative of a stronger DEP force. All cell velocities were measured frame by frame using the open-source software ImageJ and the plugin MtrackJ (imagej.net, accessed on 15 August 2018). 

## 4. Results and Discussion

### 4.1. Physical Manipulation of Single Cells

**Figure 2:**
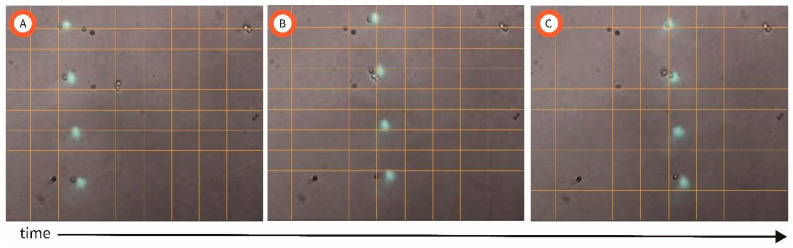
(**A**–**C**) Time lapse of the manipulation of individual *C. albicans* cells. Note how the 4 different cells follow the dot of light.

The resolution of our setup was continually improved through iterations to increase alignment of the optical path from projector to objective lens; leveling between the objective lens and the photoconductive substrate; and the minimization of mechanical vibrations. After continuous iterations of our setup, 8 µm light dots were utilized to manipulate individual *C. albicans* cells of dimensions of 5.12 ± 0.75 µm [27]. A video of this proof of concept of precise manipulation of single cells can be accessed in the Supplementary Information (Video S1). Figure 2 illustrates a time lapse of the cells following the light dots. As the light dots change position when switching slides, the individual cells follow the light pattern accordingly. Note how the number of dots and their position can be easily controlled by designing different patterns In each of the .ppt slides in the presentation deck used for experiments.

### 4.2. Effect of Pattern Shape on Cell Velocity

**Figure 3:**
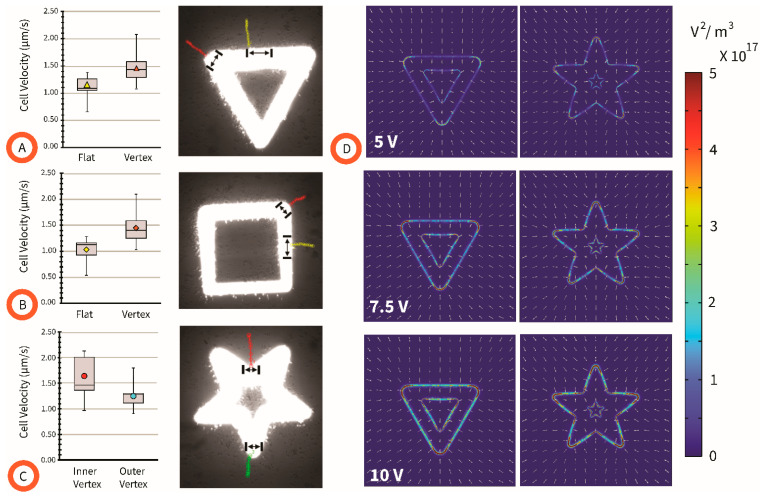
Box and whisker plots of the cell velocities (n ~30) resulting in specific locations of the shapes used in this work: (**A**) a triangle, (**B**) a square, and (**C**) a star. Cell trajectories, illustrative of the experimental results, are plotted as red or green for vertices in the shape and yellow for flats. (**D**) The distribution of ∇(*E*^2^) for the triangle and star shapes at a polarizing voltage increasing from 5 to 7.5 and 10 V. Details of the computational model shown in Figure 1C.

One potential way to affect the velocity of a targeted cell in LiDEP systems is to optimize the shape of the light pattern. Based on the literature available for conventional electrodes, we expected that the vertex of a given shape would originate a stronger DEP force when compared to the sides, or flats, of such a shape; and we hypothesized that a vertex of smaller angle would result in a stronger attractive DEP response when compared to higher angles. Results using different light pattern configurations are shown in Figure 3. All experiments reported in this section were performed using *C. albicans* cells. 

From Figure 3A,B, it is observed that the cell attraction velocity to the field gradient enabled by light is the highest at the vertex of the triangle and square shapes when compared to at its flat edges. This is reinforced by the results shown for the triangle in Figure 3D, where the field gradient is highest at the tips of the triangle. This behavior remains the same through the voltage values explored here. The voltage was swept from 5 to 10 V to accommodate the fact that ITO and illuminated a-Si are not perfect electrical conductors, and a loss of voltage is expected in the physical experiment with respect to the 10 V applied to polarize the experimental device. A further insight from Figure 3A−C is that no significant difference in cell velocity was observed with an increasing vertex angle, i.e., from outer vertex of star to vertex of square. This was unexpected but was likely due to the poor resolution of the platform at its current state, noted by the rounded vertices of each projected image independent of the shape projected. Finally, the difference between the cell velocity of those attracted to the inner and outer vertices of the star is not statistically significant, as shown in Figure 3C. This is reinforced by the results from the computational model shown in Figure 3D, which show a minimal difference between the inner and outer vertices of the star, particularly as the polarization voltage increases. In summary, the observed differences in the average cell velocity towards different regions of the projected light pattern, and the resultant electric field gradient, demonstrate the ability to tailor the strength of a cell’s positive DEP response through modifying the geometry of the projected light pattern. 

### 4.3. Effect of Pattern Color on Cell Velocity

Besides the shape of the light pattern, it is possible to change its color. As detailed by the manufacturer, DLP technology features a rotating color wheel that is synchronized with the light pattern generated by the array of micromirrors. In practice, the user can draw patterns of a particular color in Power Point, or their software of choice, which are then shined onto the LiDEP device. 

**Figure 4:**
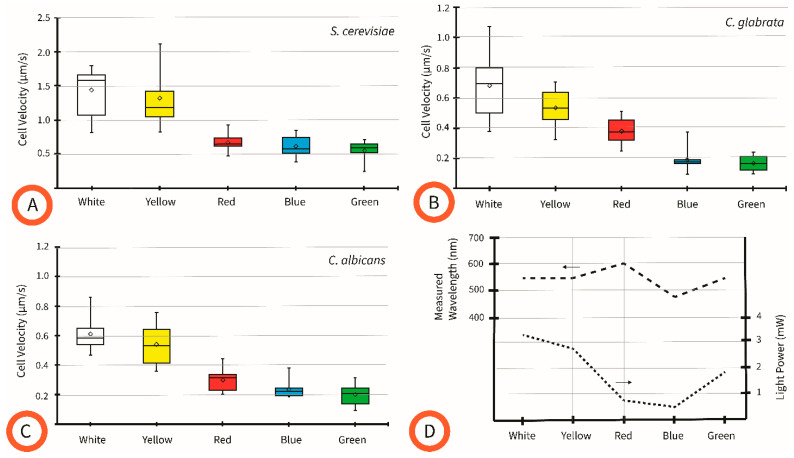
Characterization of the effect of pattern color on cell velocity. Box and whisker plots are used to display the experimental velocity data (n ~30) for three different cell spp. (**A**) *S. cerevisiae* (size 6.0 ± 0.7 µm [32]), (**B**) *C. glabrata* (size 3.24 ± 0.63 µm, as directly measured) and (**C**) *C. albicans* (size 5.12 ± 0.75 µm [27]). (**D**) The wavelength and power of the light measured from projections of the different colors used in this work.

Results are presented in Figure 4 when using the same pattern, a dot, but drawn using different colors. *S. cerevisae* displayed a higher velocity than *Candida* spp. cells across all colors tested, as evidenced by higher values in the y-axis of Figure 4A. However, there was a general trend between the color of the pattern and the observed DEP response. Dots drawn as white and yellow displayed the strongest positive DEP response, while the colors of red, blue, and green exhibited the lowest observed DEP response for all cells. Of note, the difference between white and yellow as well as those between red, blue, and green was narrow and not statistically valid in some cases, based on our data. Figure 4D shows the wavelength and intensity of the different color projections from the experimental setup. It is well known that the photoconductive properties of a-Si vary depending on the wavelength and intensity of the light projected onto it [30,31]. Smaller wavelengths and higher light intensities produce a substrate of higher electrical conductivity, and therefore generate better electrical coupling between the electrodes. Results show that the light projected by our setup when using white, yellow, and green colors (RGB values for the colors used are detailed in Section 3.1) had a wavelength of 547 nm; while blue and red had wavelengths of 473 and 600 nm, respectively. In terms of power, a projection using the white color was measured as 3.26 mW followed by yellow (2.75), green (1.84), red (0.78), and blue (0.5). In terms of wavelength, the blue pattern should have led to the highest cell velocities. However, the lower intensity of the blue projections helps interpret the lower cell velocity values. The low cell velocities in response to red patterns can be attributed to the combination of high wavelength and low intensity. White and yellow indeed provided the highest cell velocities due to their combination of wavelength and light intensity. A surprising result is that of green, where the lowest cell velocities were recorded even though its wavelength is the same as that measured for white and yellow, and its intensity is distinctively higher than red and blue. Future work will focus on identifying the reasons for such a result. Nevertheless, the observed differences in the average cell velocity when using the same shape but different colors illustrate the straightforward ability to tailor the strength of a cell’s DEP response. Changing the color drawn provides the ability to tailor the light intensity, which directly impacts the strength of the electric field gradient and ultimately the DEP response of the cell. 

### 4.4. Effect of Movement Step Size on Cell Velocity

**Figure 5:**
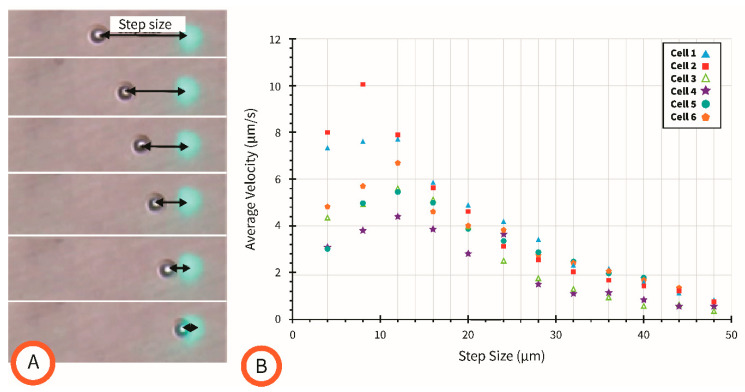
(**A**) Images from experiments showing the increasing size of the step size. (**B**) Results characterizing the relationship between step size and average velocity of a single cell. Average velocity measurements were made for six different individual cells across 12 different step sizes.

Experiments were then performed to characterize the cell velocity as a function of the distance between the targeted cell and the light pattern generating the field gradient, a dot drawn as white. Examples of the different step sizes used here are illustrated in Figure 5A. The plot of the average cell velocity as a function of step size for six different cells is shown in Figure 5B. A video of an illustrative experiment is shown as Supplementary Information. The step size in this study was the center-to-center distance between the cell and the projected light dot. All experiments in this study were performed on *C. albicans* from the same culture. Each of the six single cells studied in this work were manually isolated from the rest of the cell population using a protocol shown in the video available as Supplementary Information (Video S2). Briefly, the laser pointer feature available during a PowerPoint presentation was used to concentrate most of the cells in the field of view and manually move them away from a cell selected for study. No budding cells were included in this study. 

From Figure 5, it is seen that the average cell velocity increases with decreasing step size until the step size of the light dot is smaller than the diameter of the cell. This was expected since the strength of the DEP force originating the cell movement is a function of the electric field gradient, and the gradient of the electric field is strongest at the edges of the electrode enabled by the light dot. A deviation in the DEP response of similar cells from the same culture was also observed. The average duration of a single experiment for one given cell was a matter of seconds, so the time of the experiment is not expected to contribute to the variation in DEP response. This observed deviation is likely due to slight differences in the diameters of the cells but also may indicate slight variations in the electric properties between cells of the same population. The results of this experiment provide insightful observations on the controllability of individual cells and also show how cell velocity can be tailored to a specific magnitude based on the distance between a cell and the light pattern used to induce motion. The variation in DEP response between similar cells from the same population is expected to be a difficult challenge to overcome for precision control of cells, particularly with the intention of manipulating multiple single cells simultaneously. 

## 5. Conclusions

In this work, key details were provided for the fabrication of a LiDEP platform by hacking a commercial projector and using photoconductive electrodes using a-Si. The results for several experiments were presented that characterized the effect of different variables on the positive DEP force induced on specific cells. The ability to change the DEP response of a cell using different pattern shapes and colors was demonstrated. The effect on the distance between a single cell and a given pattern was also studied and showed that the highest achievable velocity for a cell was obtained when it was closest to the electrode edge. These results contribute to the growing knowledge about LiDEP and its potential for selected applications. Noteworthy, LiDEP still allows the user to explore the response of the target to the frequency of a polarizing AC signal like other more traditional DEP approaches. While most of the literature focuses on the physical manipulation of selected targets of different sizes, both inert and biological, using either positive or negative DEP, a few applications have been reported where LiDEP is used with flow-through microfluidics to affect the trajectory of targets due to negative DEP or to concentrate them thanks to positive DEP [33]. Hence, LiDEP remains an ideal alternative to other DEP techniques in selected applications, particularly in those that will benefit from the capability of changing the electric field gradient after the device is fabricated. 

## Data Availability

Data are contained within the article (and Supplementary Materials).

## Supplementary Materials

The following are available online at https://www.mdpi.com/article/10.3390/mi15030342/s1, Video S1: Manipulation of single cells; Video S2: Impact of step size on average cell velocity.
